# Primary adenomatoid tumor of the adrenal gland: A case report and literature review

**DOI:** 10.1097/MD.0000000000036739

**Published:** 2023-12-15

**Authors:** Hong-Feng Qi, Li-Qian Chen, Mai-Qing Yang, Xiu-Feng Li, Hai-Ning Zhang, Ke-Xin Zhang, Hong-Tao Xu

**Affiliations:** a Department of Thoracic and Cardiac Surgery, Changyi People’s Hospital, Changyi, China; b Department of Pathology, Weifang People’s Hospital (First Affiliated Hospital of Weifang Medical University), Weifang, China; c Department of Pathology, The First Affiliated Hospital and College of Basic Medical Sciences, China Medical University, Shenyang, China.

**Keywords:** adenomatoid tumor, adrenal gland, immunohistochemistry, mesothelial tumor

## Abstract

**Rationale:**

Adenomatoid tumors are rare benign tumors, mainly involving the reproductive tract, such as the epididymis in men and the uterus and fallopian tubes in women. However, a few cases can occur outside the reproductive tract. Herein, we report a rare case of a primary adenomatoid tumor of the adrenal gland.

**Patient concerns:**

A 50-year-old man underwent ultrasound examination and was found to have a right adrenal mass without elevated blood pressure, weakness after fatigue, frequent nocturnal urination urgency, pain, or a history of hematuria. The patient’s general health was normal. Computed tomography revealed a polycystic mixed-density lesion in the right adrenal region, approximately 7.3 × 4.5 cm in size.

**Diagnoses:**

Based on the clinical information, morphological features, and immunohistochemistry results, a pathological diagnosis of primary adenomatoid tumor of the adrenal gland was made.

**Intervention:**

Excision of the right adrenal gland and tumor through the 11 ribs.

**Outcomes:**

The patient’s postoperative course was uneventful.

**Lessons:**

Preventing misdiagnosis adenomatoid tumors with other types of adrenal gland tumors or metastatic tumors is imperative. Morphological and immunohistochemical features can help diagnose primary adenomatoid tumors of the adrenal gland.

## 1. Introduction

Adenomatoid tumors (ATs) are rare benign tumors of mesothelial origin that often occur in the reproductive tract, more common in the epididymis in men and in the uterus and fallopian tubes in women. Nonetheless, few cases can occur outside the reproductive tract.^[1]^ Primary ATs outside of the reproductive tract, particularly in the adrenal glands, are extremely rare. To our knowledge, only 40 cases of adrenal AT have been reported to date in published literature in English. Herein, we report a case of adrenal AT in a 50-year-old male patient. The features of adrenal AT were assessed and summarized.

## 2. Case presentation

### 2.1. Ethical approval

This study was approved by the Institutional Review Board of China Medical University for Human Studies (approval number: LS [2021] 009). Written informed consent was obtained from the patient for the publication of this case report and the accompanying images. This study was conducted in accordance with the principles of the Declaration of Helsinki.

### 2.2. Clinical history

**Figure 1.**
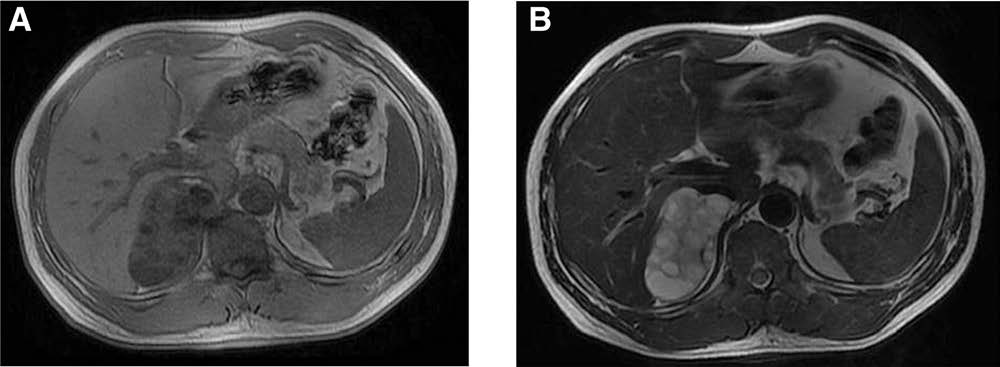
Computed tomography of the abdomen. (A and B), Computed tomography scan showing a polycystic mixed-density lesion in the right adrenal region, approximately 7.3 × 4.5 cm in size.

A 50-year-old man with a chief complaint of right adrenal gland mass during a physical examination before 5 days was admitted to our hospital for further treatment. The patient underwent an ultrasound examination at a local hospital 5 days prior, and a right adrenal mass was found without elevated blood pressure, weakness after fatigue, nocturnal urination frequency, urgency, pain, or a history of hematuria. Subsequent computed tomography revealed a polycystic mixed-density lesion in the right adrenal region, approximately 7.3 × 4.5 cm in size. Enhancement revealed that the cyst wall of the lesion was uneven in thickness. The plain computed tomography value was 49 HU, which increased after enhancement (73 HU) (Fig. 1). Preoperatively, the lesion was presumed to be a space-occupying lesion in the right adrenal region, following which a surgical excision of the right adrenal gland and tumor was performed through the 11 ribs. The patient did not receive any postoperative therapy and recovered well after surgery. Follow-up at 6 years did not reveal any evidence of recurrence or other metastatic diseases.

### 2.3. Immunohistochemical staining

The resected specimens were fixed with 10% neutral-buffered formalin, embedded in paraffin blocks, and sliced into 4 μm-thick sections. The sections were then stained with hematoxylin and eosin for histological assessment. Some tumor sections were immunostained with ready-to-use primary antibodies against broad-spectrum cytokeratin (CK), vimentin, calretinin, Wilms tumor gene-1 (WT-1), D2–40, Paird box 8, synaptophysin, chromogranin-A, inhibin-α, S-100, Melan-A, HMB45, CD34, and Ki-67 (Maixin, Fuzhou, China). Subsequent detection was performed using the streptavidin-peroxidase method after incubation with the primary antibody. Positive and negative controls were used as appropriate.

### 2.4. Morphological and immunohistochemical findings

Grossly, the tumor was approximately 9 × 5 cm in size with a smooth surface and light yellow in color. The cut section of the tumor was multilocular.

**Figure 2.**
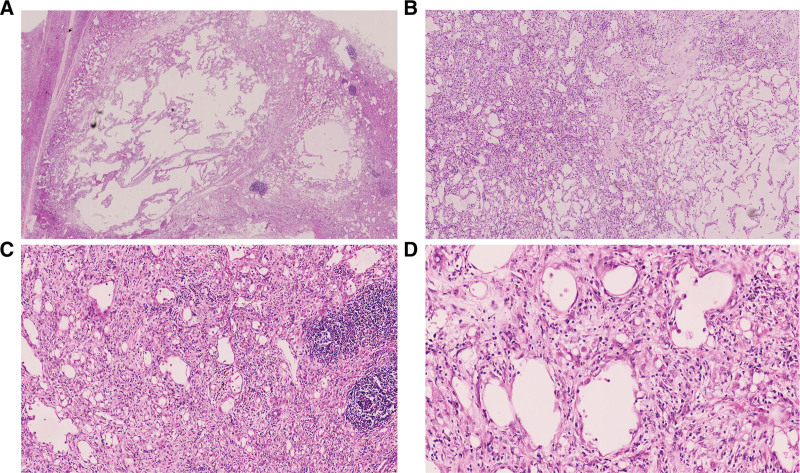
Histological features associated with adrenal adenomatoid tumor. (A) The tumor tissues present a polycystic structure and expansive growth pattern; however, infiltration into the normal adrenal tissue is observed in some regions (hematoxylin and eosin, × 15). (B) The tumors consisted of different patterns, including irregular glandular, microcystic, and cystic structures (hematoxylin and eosin, × 50). (C) Lymphocytic infiltration observed in the stroma with a few lymphatic follicles formation in some regions (hematoxylin and eosin, × 100). (D) The tumor cells were mostly flat or columnar, while some cells exhibiting signet ring cell-like morphology. The nuclei were not heterotypic, without clear mitotic figures or tumor necrosis. (hematoxylin and eosin, × 200).

Microscopically, the tumors exhibited different growth patterns, including irregular glandular, microcystic, and cystic structures. The tumor cells were mostly flat or columnar; some tumor cells exhibited a signet ring cell-like morphology. The nuclei were not heterotypic, without clear mitotic figures or tumor necrosis. Tumor cells infiltrate the normal adrenal tissue in some areas. Lymphocyte infiltration was observed in the stroma, with a few lymphatic follicles in some regions (Fig. 2).

**Figure 3.**
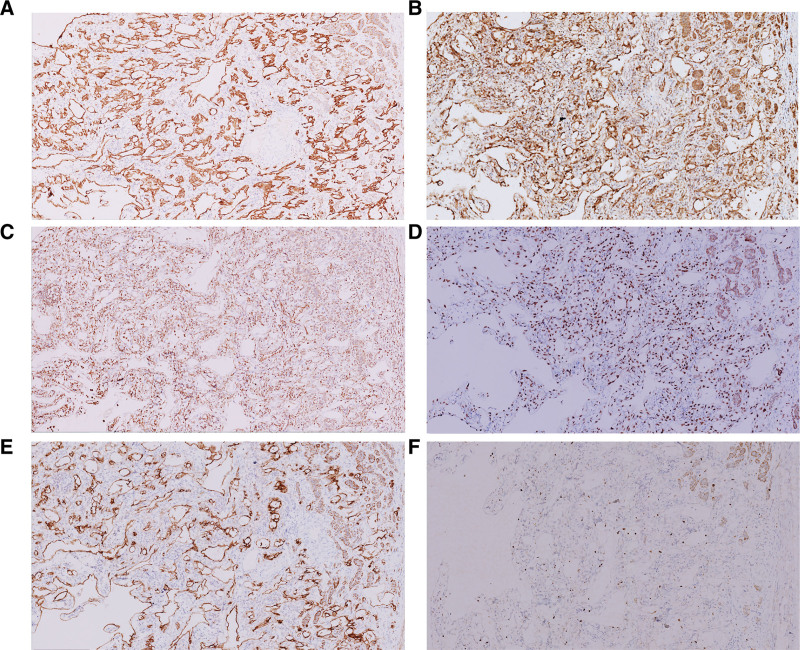
Immunohistochemistry of the adrenal adenomatoid tumor. (A) Tumor cells diffuse positive for CK (× 100). (B) Tumor cells positive for calretinin (× 100). (C) Tumor cells positive for vimentin (× 100). (D) Tumor cells positive for WT-1 (× 100). (E) Tumor cells positive for D2–40 (× 100). (F) The Ki-67 index of tumor cells was 5% (× 100). CK = cytokeratin WT-1, Wilm’s tumor gene-1.

Immunohistochemically, the tumor cells were positive for CK, calretinin, D2-40, WT-1, and vimentin, and negative for Paird box 8, synaptophysin, chromogranin-A, inhibin-α, S-100, Melan-A, HMB45, and CD34. The Ki-67 index was < 5% (Fig. 3).

## 3. Discussion

Based on the clinical findings, morphological features, and immunohistochemical results, a pathological diagnosis of primary adrenal AT was made.

AT is a rare benign tumor that originates from the mesothelial cells and typically occurs in the epididymis, uterus, fallopian tubes, ovaries, and other areas. Primary adrenal AT is extremely rare. Evans et al^[2]^ reported the first case of primary adrenal AT in 1988. To our knowledge, in patients with adrenal AT, including the present case, only 41 patients with adrenal AT have been reported to date in the English medical literature. Table 1 summarized the clinicopathological features of the patients. Primary adrenal AT had been reported in 2 women and 39 men aged between 22 and 64 years (mean age: 40.0 years), indicating a sex predilection for men. The diameters of the tumors ranged from 0.5 to 19 cm (mean: 4.5 cm), except for 1 microscopical tumor and 1 case without description. The tumor location showed no preference for the left or right adrenal gland. Of the 41 reported cases, 21 and 19 cases were reported in the right and left adrenal glands, respectively (not documented in 1 patient). Except for 4 patients who were not available, lymphocytic infiltration or aggregation was observed in 27 patients and not reported in 10 patients.^[2–31]^

**Table 1:** Clinicopathological characteristics of patients with primary adenomatoid tumor of the adrenal gland.

Case	Symptoms	Year	Sex	Age	Size (cm)	Site	Lymphocytes infiltration/aggregation	Follow-up
1^[2]^	IRF	1988	M	36	11.0	L	Not stated	8 mo
2^[3]^	IRF	1990	M	24	1.1	L	Not stated	6 mo (died of pulmoray carcinoid)
3^[4]^	IRF	1990	M	44	3.2	L	Yes	177 mo
4^[5]^	IFA	1996	M	49	1.3	R	Not available	Found at autopsy
5^[5]^	IFA	1996	M	57	3.8	L	Not available	Found at autopsy
6^[5]^	IFA	1996	F	50	0.5	R	Not available	Found at autopsy
7^[5]^	IRF	1996	M	40	6.0	L	Not available	Not stated
8^[6]^	IFA (AIDS)	1997	M	34	3.0	R	Not stated	Found at autopsy (died of acute bilateral pneumonia)
9^[7]^	IRF	1999	M	28	9.0	R	Not stated	16 mo
10^[8]^	IRF	2000	M	54	6.5	L	Yes	Not stated
11^[9]^	IFS (rectal adenocarcinoma)	2003	M	37	3.1	L	Yes	40 mo
12^[10]^	IRF	2003	M	31	3.2	R	Yes	Not stated
13^[10]^	IRF	2003	M	31	3.5	Not stated	Yes	50 mo
14^[10]^	IFA	2003	M	64	1.2	L	Yes	Found at autopsy
15^[10]^	IRF	2003	M	51	3.0	R	Yes	Not stated
16^[11]^	IRF	2003	M	33	1.7	L	Not stated	Not stated
17^[12]^	IRF	2004	M	42	19.0	L	Not stated	3 yr
18^[13]^	IRF	2005	M	46	Microscopical	L	Yes	Not stated
19^[13]^	IRF	2005	M	33	1.7	L	Yes	Not stated
20^[13]^	IRF	2005	M	33	4.2	R	Yes	1 yr
21^[14]^	IRF	2005	M	54	3.6	R	Not stated	1 yr
22^[15]^	IFA	2005	M	30	3.0	L	Yes	Found at autopsy (acute coronary thrombosis)
23^[16]^	IRF	2005	M	42	2.5	L	Not stated	Not stated
24^[17]^	IRF	2008	M	47	7.0	R	Yes	Not stated
25^[17]^	IRF	2008	M	52	5.5	R	Yes	Not stated
26^[18]^	IRF	2008	M	26	15.0	R	Not stated	Not stated
27^[19]^	IRF	2009	M	39	5.5	R	Yes	Not stated
28^[20]^	IRF	2009	M	60	11.0	R	Yes	Not stated
29^[21]^	IRF	2010	M	29	4.0	R	Yes	Not stated
30^[22]^	IRF	2010	M	51	Not stated	L	Yes	Not stated
31^[23]^	IRF	2010	M	44	17.0	L	Not stated	3 mo
32^[24]^	IRF	2011	M	22	2.5	R	Yes	7 mo
33^[25]^	IRF	2011	M	24	3.6	L	Yes	6 mo
34^[26]^	IRF	2013	M	32	4.0	L	Yes	2.5 yr
35^[27]^	IRF	2013	M	62	3.0	R	Yes	8 mo
36^[28]^	IRF	2015	M	40	5.5	R	Yes	1 yr
37^[29]^	IRF	2016	F	30	8.0	R	Yes	4 yr
38^[30]^	IRF	2020	M	28	4.8	R	Yes	Not stated
39^[31]^	IRF	2021	M	30	3.5	R	Yes	21 mo
40^[31]^	IRF	2021	M	31	8.0	L	Yes	8 mo
41 (our case)	IRF	2023	M	50	9.0	R	Yes	6 yr

F = female, IFA = incidental finding during autopsy, IFS = incidental finding during surgery for unrelated reasons, IRF = incidental radiographic finding, L = left adrenal gland, M = male, R = right adrenal gland.

Generally, adrenal ATs appear as smooth and clear solitary nodules, some of which can compress the surrounding adrenal tissue with a soft texture and a solid or cystic appearance. Microscopically, the tumor is often composed of multiple glandular, microcystic, and cystic regions and often forms fissured and mutually anastomotic cavities lined with flattened endothelioid cells or eosinophilic epithelioid cells. Prominent vacuolated ring-like cells with no nuclear atypia or mitotic figures may also be present in varying numbers. Lymphocyte infiltration and even the formation of lymphatic follicles can be observed in the stroma without any proliferative response to the fibrous connective tissue. Tumor cells express mesothelial markers such as CK, calretinin, D2–40, WT-1, and vimentin. The morphological and immunohistochemical results in the present case were consistent with these features.

Because mesothelial cells are not present in the adrenal gland, it is currently believed that adrenal adenomatoid tumors may originate from multipotent mesenchymal cells associated with the Müllerian duct. Distinguishing this tumor from other primary or metastatic tumors with similar histological features, such as lymphangiomas, hemangiomas, metastatic adenocarcinomas, and epithelioid hemangioendotheliomas, is imperative. Both lymphangiomas and hemangiomas exhibit cystic or fissure-like structures. Moreover, tumor cells of lymphangioma and hemangioma are positive for immunohistochemical markers, such as CD31 and CD34, and negative for mesothelial markers such as calretinin, D2–40, and WT-1, which is helpful in distinguishing them from AT. Metastatic adenocarcinoma is generally bilateral and multiple, and it is often difficult to distinguish between metastatic adenocarcinomas, signet ring cell carcinoma in particular, and invasive glandular-like structures and signet ring-like cell regions in AT. Adenocarcinoma cells exhibit obvious atypia and are often accompanied by tumor necrosis. The cytoplasm of the signet ring cells is rich in mucus, whereas the signet ring-like cells of AT exhibit no nuclear atypia or cytoplasmic mucus. In addition, adenocarcinoma cells are often positive for CEA and negative for mesothelial markers. Epithelioid hemangioendothelioma often shows an unclear vascular structure and round or polygonal tumor cells with abundant cytoplasm. Visible lumen or vacuole formation was observed within the cytoplasm of the tumor cells. Tumor cells express endothelial cell markers such as CD34, CD31, and ERG, and negative for mesothelial markers. A complete surgical resection of adrenal AT is curative and feasible and does not require postoperative adjuvant treatment. All patients with adrenal ATs underwent surgery alone. Definitive histopathological prognostic factors of adrenal AT have not been fully clarified because of its rarity. No local recurrence or metastasis has been reported in patients with adrenal ATs. In our case, the patient underwent surgery alone, and no recurrence was reported at 6 years postoperatively.

In summary, we report a rare case of primary adrenal AT treated with complete surgical resection of the tumor. A meticulous assessment of histological features and immunohistochemical examination will aid in an accurate and effective diagnosis.

## Acknowledgements

We would like to thank Editage (www.editage.cn) for English language editing.

## Author contributions

**Conceptualization:** Hong-Tao Xu.

**Methodology:** Hong-Feng Qi, Li-Qian Chen, Xiu-Feng Li, Hai-Ning Zhang, Ke-Xin Zhang.

**Writing – original draft:** Hong-Feng Qi, Mai-Qing Yang, Hong-Tao Xu.

**Writing – review & editing:** Mai-Qing Yang, Hong-Tao Xu.
